# EXPERIENCE OF PLASMAPHERESIS PERFORMED WITH CRRT MONITORS BY ICU STAFF

**DOI:** 10.1186/2197-425X-3-S1-A57

**Published:** 2015-10-01

**Authors:** M Yagüe-Huertas, A Martín-Vivas, A Leal-Micharet, Á Gabán-Díez, AI González-Jiménez, J Ferrero-Calleja, R Ruiz-de Luna, N de la Calle-Pedrosa, A Hernández-Tejedor, M Sigcha-Sigcha, I Temprano-Gómez, A Algora-Weber

**Affiliations:** Hospital Universitario Fundación Alcorcón, Intensive Care Medicine, Alcorcón, Spain

## Introduction

Plasmapheresis (PF) is an extracorporeal blood purification technique designed to remove large molecular weight particles from plasma. There is growing evidence on the benefit of this technique in different clinical diseases that would deserve intensive care.

## Objectives

To report our experience in PF performed with CRRT monitors (Prismaflex® System, Gambro) with a filter membrane, which allows the removal of particles up to 3x10^6^ Da. To analyze the characteristics of our patients, the technique and its complications.

## Methods

Assessment of all the PF sessions carried out in a 12-bed medical ICU in Spain (October 2013-February 2015). Hemodynamic data were measured before and during each session. Haematological and biochemical parameters were analyzed before, at the end of each session, and thereafter following a recovery period (13.9 ± 0.5 hours). Statistical paired analysis was made by Wilcoxon test.

## Results

A total of 49 sessions were performed in 5 patients with neurological disease. 80% of our patients were female. Median age: 42 (IQR 35-71) years. Median number of sessions in each cycle: 4 (range 3-6). Plasma volume exchange ratio was 1:1. Replacement fluid used was Hemosol B0 ^TM^ (Gambro) with addition of albumin (to achieve a concentration of 3-4 g/dL) and KCl (4.1 ± 0.3 mmol/L). Session mean duration: 2.9 ± 0.1 hours.

During PF, a decrease (p < 0.05) in systolic BP (124 ± 17 to 113 ± 19 mmHg) and diastolic BP (68 ± 11 to 62 ± 10 mmHg) was observed. Nevertheless, norepinephrine up to 0.27 mcg/kg/min was only necessary in 10% (5/49) of the sessions. Main significant analytical results in Table 1.

**Table 1:** PF. Mean ± SD; Median(IQR).

	BEFORE PF	Before vs after	AFTER PF	After vs recovery	RECOVERY	Before vs recovery
Leukocytes (10^3^ /µL)	9.4 ± 2.3	P < 0.05	14.8 ± 5.4	P < 0.05	10.6 ± 3.3	P < 0.05
Haemoglobin (g/dL)	10.8 ± 1.8	P < 0.05	11.5 ± 2.1	P < 0.05	10.8 ± 1.9	P= 0.21
Platelets(10^3^ /µL)	221.8 ± 85.3	P = 0.49	213.1 ± 86.1	P < 0.05	209.7 ± 89	P < 0.05
Prothrombin Act. (%)	97.7 ± 12.1	P < 0.05	53.2 ± 12.4	P < 0.05	81.5 ± 11	P < 0.05
Fibrinogen (mg/dL)	232.2 ± 107.7	P < 0.05	100.1 ± 57.2	P < 0.05	164.4 ± 58.5	P < 0.05
Magnesium (mg/dL)	2 (1.9-2.2)	P < 0.05	1.9 (1.7-2.1)	P < 0.05	2 (1.8-2.1)	P = 0.16
Phosphorus (mg/dL)	3.2 (2.6-3.4)	P < 0.05	2.7 (2.4-2.9)	P < 0.05	3.1 (2.6 - 3.4)	P = 0.42
Ionized Calcium (mmol/L)	1.15(1.1-1.17)	P < 0.05	1.1(1.07-1.13)	P < 0.05	1.14(1.1-1.16)	P = 0.68
Albumin (g/dL)	3.2 ± 0.5	P < 0.05	3.8 ± 0.4	P < 0.05	3.3 ± 0.6	P= 0.13

Transient significant leukocytosis with no signs of infection developed after PF. This was due to an increase in the neutrophils count (6.4 ± 2.5 to 12.4 ± 5.3 cells 10^3^/µL,p < 0.05); lymphocytes trend to decrease although without reaching statistical significance (1.9 ± 1.5 to 1.6 ± 1 cells 10^3^/µL,p= 0.09). Although serum Sodium and Chloride varied significantly with PF, their levels remained within normal ranges, whereas serum Potassium remained stable due to replacement fluid concentration administered.

Red blood cell transfusion was accomplished in 8.2%(4/49), platelets transfusion in 2%(1/49) and fresh-frozen plasma transfusion in 6%(3/49) of the sessions. A mean fibrinogen concentrate dose of 1.9 ± 1.2g. was administrated in 59.2%(29/49) of the sessions. Electrolytes disturbances were corrected based on medical criteria.

Complications detected were coagulation of extracorporeal system(n = 1), catheter thrombosis(n = 1) and femoral catheter contamination(n = 1) due to indwelling catheter urinary tract infection. No major bleeding, or therapy-related deaths were registered.

## Conclusions

PF is a safe therapy performed with CRRT monitors by ICU staff. Hemodynamic, hematological and biochemical changes occur with no related symptoms whether early appropriate adjustment is established. Close electrolytes monitoring for prompt correction is mandatory.

